# Impact of Phosphorylcholine Expression on the Adherence and Invasion of *Streptococcus pyogenes* to Epithelial Cells

**DOI:** 10.3390/microorganisms10030527

**Published:** 2022-02-28

**Authors:** Hiroyuki Iuchi, Junichiro Ohori, Hisahiro Matsuzaki, Takeshi Tokushige, Sakiko Toge, Masaru Yamashita

**Affiliations:** Department of Otolaryngology, Head and Neck Surgery, Kagoshima University Graduate School of Medical and Dental Sciences, Kagoshima 890-8520, Japan; juno@m3.kufm.kagoshima-u.ac.jp (J.O.); m_hisa1231@yahoo.co.jp (H.M.); t_tokushige1992@outlook.jp (T.T.); peony220s@outlook.jp (S.T.); yamashita@kufm.kagoshima-u.ac.jp (M.Y.)

**Keywords:** phosphorylcholine, *Streptococcus pyogenes*, PAF-R, *emm* genotype

## Abstract

Phosphorylcholine (PC) is a structural component of various pathogens and is involved in bacterial adhesion via the platelet-activating factor receptor (PAF-R). In this study, we investigated how PC expression affects cell adhesion and invasion of *Streptococcus pyogenes* (*S. pyogenes*). Eight clinical strains of *S. pyogenes* were cultured, and PC expression was measured using fluorescence-activated cell sorting. Bacterial adherence and invasion were examined using Detroit 562 cells. An anti-PC-specific monoclonal antibody (TEPC-15) was used to inhibit bacterial PC, and a PAF-R antagonist (ABT-491) was used to inhibit cellular PAF-R. The *emm* gene was amplified by the polymerase chain reaction with the standard primers. The level of PC expressed on the *S. pyogenes* surfaces differed in each strain and differed even in the same *emm* genotype. Adherence assay experiments showed a significant negative correlation between TEPC-15 and ABT-491 inhibitory effects and PC expression in *S. pyogenes*. Similarly, intracellular invasion assay experiments showed a significant negative correlation between TEPC-15 and ABT-491 inhibitory effects and PC expression in *S. pyogenes*. This study suggests that *S. pyogenes* is involved in cell adhesion and invasion by PC.

## 1. Introduction

Recurrent tonsillitis is a common disease encountered by otolaryngologists [1]. Viruses are the most common causes of recurrent tonsillitis, followed by bacteria, including *Streptococcus pyogenes* (*S. pyogenes*), *Haemophilus influenzae*, and *Streptococcus pneumoniae* [2]. *S. pyogenes* is a species of Gram-positive bacteria that causes respiratory tract infections with mild to modest disease (tonsillitis and pharyngitis) and invasive and potentially life-threatening diseases (cellulitis, necrotizing fasciitis, and toxic shock syndromes) [3,4]. Mortality from lethal *S. pyogenes* remains high in developed and developing countries [5]. In addition, there are reports of *S. pyogenes* outbreaks in communities and hospitals [6]. Early diagnosis is required, and a recent study reported that marker genes (*spyCEP*, *ssa*, *sic*, *sdaB*, *speG*) can be used as a rapid diagnostic tool for *S. pyogenes* [7].

One of the first basic stages of *S. pyogenes* etiology is epithelial adhesion and colonization [8]. The production of reactive oxygen species by pathogen-infected cells is often associated with high levels of cell death, and *S. pyogenes* induce apoptosis in infected human epithelial cells [4]. Moreover, *S. pyogenes* induce the transcription of the interleukin (*IL)-1a*, *IL-1b*, *IL-6*, and *IL-8* genes and the release of prostaglandin E2 [9].

The surface of *S. pyogenes* cells incorporates a number of proteins adhesins (e.g., pili, Sfb1 / PrtF1, and M protein) that allow *S. pyogenes* to colonize distinct tissue sites [10]. The prominent M protein is important for the attachment of *S. pyogenes* to keratinocytes in skin infections [4]. M protein, a cell surface protein that is a major pathogenic determinant of *S. pyogenes*, is encoded by the *emm* gene [11]. The *emm* genotype of *S. pyogenes* has more than 170 genotypes and 750 subtypes, and the genotype distribution varies by country or region [11,12].

In contrast, phosphorylcholine (PC) involvement in *S. pyogenes’* cell adhesion is unknown. PC is a structural component of many bacteria, such as *Streptococcus pneumoniae* and non-typeable *Haemophilus influenzae* [13,14]. Bacterial PC binds to the platelet-activating factor receptor (PAF-R) on the cell surface and plays an important role in *Streptococcus pneumoniae* and non-typeable *Haemophilus influenzae* [13,14]. However, there is no report on the relationship between the PC expression of *S. pyogenes* and PAF-R or *emm* gene.

In this study, we investigated the effect of bacterial surface PC expression on the adhesion and invasion in epithelial cells in *S. pyogenes.* We also investigated the relationship between PC expression and the *emm* gene.

## 2. Materials and Methods

### 2.1. Bacteria Culture

From March 2019 to December 2020, *S. pyogenes* were collected from the oropharynx of patients with recurrent tonsillitis (age 24–42 years) at Kagoshima University Hospital. Bacteria were stored in skim milk containing glycerol at −80 °C and incubated overnight in a 5% CO_2_ incubator at 37 °C in sheep blood agar (Nissui Pharmaceutical Co., Ltd., Tokyo, Japan) the day before use. After washing with 0.5% bovine serum albumin-phosphate buffered saline (PBS), we measured the optical density at 580 nm and determined the colony-forming units (CFU). The concentration of *S. pyogenes* was adjusted to 1.0 × 10^8^ CFU/mL, as previously described [8].

### 2.2. Cell Culture

Detroit 562 cells (CCL-138; ATCC, Manassas, VA, USA), which are human pharyngeal carcinoma epithelial cells, were cultured in minimal essential medium (Nacalai Tesque Inc., Kyoto, Japan) in a 5% CO_2_ incubator at 37 °C, as previously described [9]. The cells were harvested using trypsin (final concentration, 0.02%) and ethylenediaminetetraacetic acid (final concentration, 0.02%; Nacalai Tesque). Thereafter, 2 × 10^4^ viable cells were placed per well on a 96-well BD Falcon tissue culture plate and cultured in a 5% CO_2_ incubator at 37 °C for 48 h. It was confirmed that the cells were sufficiently cultured in the culture plate.

### 2.3. PC Expression of Streptococcus pyogenes

PC expression was measured using mean fluorescence intensity (MFI) by fluorescence-activated cell sorting (FACS; CytoFLEX, Beckman Coulter, Tokyo, Japan). Bacteria cultured overnight on a blood agar plate were adjusted to 1.0 x 10^8^ CFU/mL with PBS. Then, PC-specific monoclonal mouse IgA antibody (TEPC-15; Sigma-Aldrich, St. Louis, MO, USA) was incubated at 4 °C for 4 h. Finally, the bacteria were incubated with fluorescein isothiocyanate-labeled goat anti-mouse antibody (1:50 dilution, KPL, Gaithersburg, ML, USA) at 20 °C for 30 min.

### 2.4. emm Genotypes of S. pyogenes

The *emm* gene encoding the M protein was performed according to the recommended protocol by the Center for Disease Control and Prevention (http://www.cdc.gov/ncidod/biotech/strep/protocols.html, accessed on 16 April 2020, with minor modifications, as previously described [15].

### 2.5. Adherence Assay

Bacteria were incubated on a sheep blood agar plate overnight at 37 °C in a 5% CO_2_ incubator. Bacteria (1.0 × 10^5^ CFU/mL) were administered to a 96-well plate and adhered to at 37 °C for 2 h in a 5% CO_2_ incubator. Each well was washed 10 times with 200 μL PBS to remove bacteria that did not adhere to the cells. Then, it was treated with 100 μL saponin at 37 °C for 15 min in a 5% CO_2_ incubator, and 100 μL of solution from each well was seeded on sheep blood agar plates. After incubation for 12 h, it was counted as a control. To investigate the effect of PC-specific IgA on bacterial adhesion, the number of colonies was counted. Bacteria were treated with TEPC-15 (1 μg/mL) at 37 °C for 1 h in a 5% CO_2_ incubator to investigate the effect of PC on bacterial adhesion. Furthermore, PAF-R expressed on the surface of Detroit 562 cells was blocked with a 10 μg/mL PAF-R antagonist (ABT-491; Cayman Chemical, Ann Arbor, MI, USA) at 37 °C for 1 h in a 5% CO_2_ incubator.

### 2.6. Intracellular Invasion Assay

Bacteria were incubated on a sheep blood agar plate overnight at 37 °C in a 5% CO_2_ incubator. Bacteria (1.0 × 10^8^ CFU/mL) were administered to a 96-well plate and invaded at 37 °C for 6 h in a 5% CO_2_ incubator. Then, gentamicin (200 μg/mL) was added to each well at 37 °C in a 5% CO_2_ incubator for 1 h. Each well was washed 10 times with 200 μL of PBS. It was then treated with 100 μL of saponin at 37 °C for 15 min in a 5% CO_2_ incubator, and 100 μL of the solution from each well was seeded on sheep blood agar plates. After incubating for 12 h, the number of colonies was counted. In addition, as in the adherence assay, bacteria were treated with TEPC-15, and cells were treated with ABT-491 to investigate their effects.

### 2.7. Statistical Analyses

Statistical analyses were conducted using SPSS for Mac software (version 22.0; IBM Corp., Armonk, NY, USA). The data were statistically analyzed using the unpaired Student’s t-test and Pearson’s correlation coefficient (cross-reaction data). The values are presented as means ± standard deviations. The level of significance was set at *p* ≤ 0.05.

## 3. Results

### 3.1. PC Expression and emm Genotype

In *S. pyogenes*, PC was expressed in all strains, but the intensity differed in each strain (Table 1). In this study, we detected four strains of genotype *emm*75 and one strain of each genotype *emm*89, *emm*28, *emm*12, and *emm*11 (Table 1). Furthermore, PC expression tended to be high in *emm*75 strains, and PC expression was different even in the same *emm*75 strains (Table 1).

PC expression was different among the bacterial strains. In addition, PC expression was different, even with the same *emm* genotype.

### 3.2. Inhibitory Effects of TEPC-15 on Bacterial Adherence

**Figure 1:**
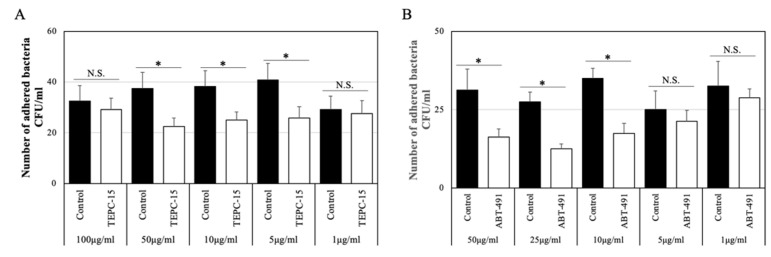
Adhesion inhibitory effect at concentrations of TEPC-15 and ABT-491. In TEPC-15, bacterial adhesion was significantly suppressed at 50, 10, and 5 μg/mL (*p* < 0.05). (**A**) In ABT-491, bacterial adhesion was significantly suppressed at ≥10 μg/mL (*p* < 0.05). (**B**) Error bars indicate the standard deviation of the mean (*n* = 5). * *p* ≤ 0.05.

As shown in Figure 1A, the number of adhered bacteria (strain No. 7) treated with TEPC-15 at 50, 10, and 5 μg/mL significantly decreased (*p* < 0.05), but not with TEPC-15 at 100 and 1 μg/mL. Based on these results, the inhibitory effects of 10 μg/mL of TEPC-15 or mouse IgA antibody as a control were used on the adherence and intracellular protocol.

### 3.3. Inhibitory Effects of ABT-491 Dose on Bacterial Adherence

As shown in Figure 1B, the number of adhered bacteria (strain No. 7) treated with ABT-491 at 50, 25, and 10 μg/mL significantly decreased (*p* < 0.05), but not with ABT-491 at 5 and 1 μg/mL. Based on these results, the inhibitory effects of 10 μg/mL of ABT-491 were used on the adherence and intracellular protocol.

### 3.4. Effects of TEPC-15 and ABT-491 on Bacterial Adherence

**Figure 2:**
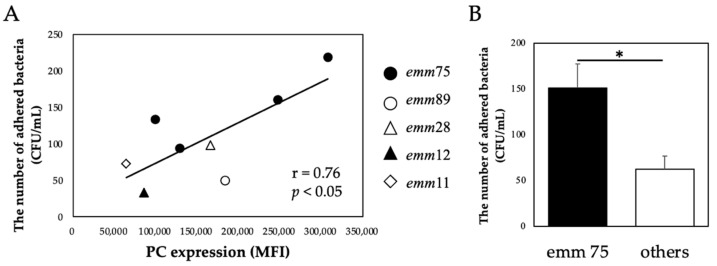
PC expression and bacterial adhesion. In *Streptococcus pyogenes*, PC expression and cell adhesion showed a positive correlation (*n* = 10, r = 0.76, *p* < 0.05). (**A**) Furthermore, *emm*75 had higher adhesiveness than the other *emm* genotypes. (**B**) Error bars indicate the standard deviation of the mean (*n* = 5). * *p* < 0.05. MFI, mean fluorescence intensity; PC, phosphorylcholine.

**Figure 3:**
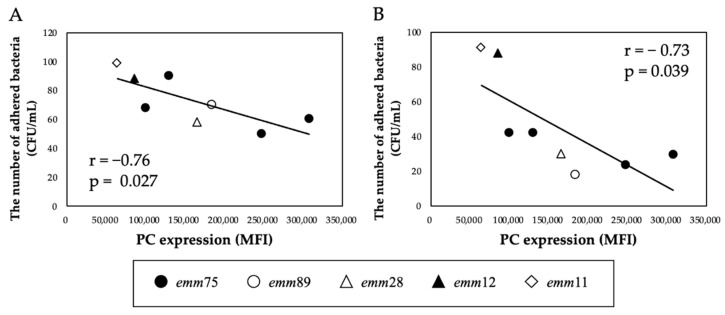
Inhibitory effect of TEPC-15 and ABT-491 on the adhesion of *Streptococcus pyogenes.* Inhibition of adhesion by pretreatment with TEPC-15 (*n* = 5, r = −0.76, *p* = 0.027) (**A**) and ABT-491(*n* = 5, r = −0.73, *p* = 0.039) (**B**) showed a negative correlation with PC expression in *Streptococcus pyogenes*. MFI, mean fluorescence intensity; PC, phosphorylcholine.

There was a significant positive correlation between the PC expression of each bacteria and the number of bacteria adhering to the cells (r = 0.76, *p* < 0.05) (Figure 2A). Furthermore, the number of adhered *emm*75 was significantly higher than that of other *emm* genotypes (*p* < 0.05) (Figure 2B). There was a significant negative correlation between the PC expression of each bacteria and the number of bacteria adhering to the cells after pretreatment with TEPC-15 (r = −0.76, *p* = 0.03) (Figure 3A). Moreover, a negative correlation was found with ABT-491 (r = −0.73, *p* = 0.04) (Figure 3B).

### 3.5. Effects of TEPC-15 and ABT-491 on Bacterial Invasion

**Figure 4:**
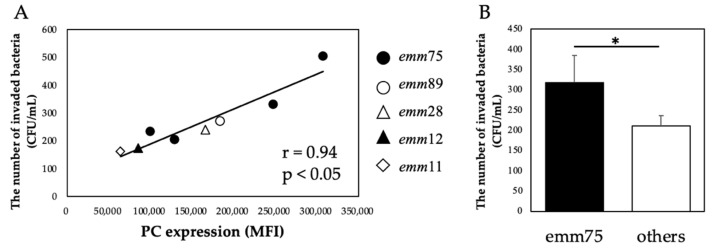
PC expression and bacterial invasion. In *Streptococcus pyogenes*, PC expression and cell invasion showed a positive correlation (r = 0.94, *p* < 0.05). (**A**) Furthermore, *emm*75 was more invasive than the other types. (**B**) Error bars indicate the standard deviation of the mean (*n* = 5). * *p* < 0.05. MFI, mean fluorescence intensity; PC, phosphorylcholine.

**Figure 5:**
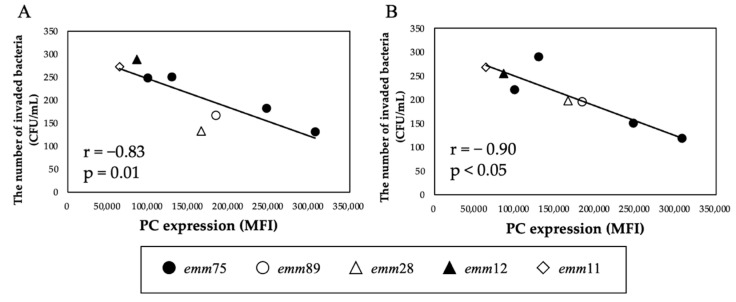
Inhibitory effects of TEPC-15 and ABT-491 on cell invasion of *Streptococcus pyogenes.* Inhibition of invasion by pretreatment with TEPC-15 (*n* = 5, r = −0.83, *p* = 0.01) (**A**) and ABT-491 (*n* = 5, r = −0.90, *p* < 0.05) (**B**) showed a negative correlation with phosphorylcholine expression in *Streptococcus pyogenes*. MFI, mean fluorescence intensity.

There was a significant positive correlation between the PC expression of each bacteria and the number of bacteria invading the cells (r = 0.94, *p* < 0.05) (Figure 4A). Furthermore, when comparing the number of invaded *emm*75 with other *emm* types, the number of invaded *emm*75 was significantly higher (*p* < 0.05) (Figure 4B). There was a significant negative correlation between the PC expression of each bacteria and the number of bacteria adhering to the cells after pretreatment with TEPC-15 (r = −0.83, *p* = 0.01) (Figure 5A). Moreover, a negative correlation was found with ABT-491(r = −0.90, *p* < 0.05) (Figure 5B).

## 4. Discussion

This study showed that PC expression was found in *S. pyogenes*, and PC expression was different even for the same *emm* genotype. High bacterial PC expression also increased the number of bacteria adhered to the cells. In addition, treatment with TEPC-15 and ABT-491 further suppressed adhesion and invasion of bacteria with high PC expression.

The existence of the PC was first discovered in *Streptococcus pneumoniae* by Tomasz in 1967 [16]. PC are small hapten moieties that bind to teichoic acid in the cell walls of several Gram-positive bacteria, including *Streptococcus pneumoniae* [17]. In addition, the phase variation of PC expression provides a mechanism for *Haemophilus influenzae* to display a variety of phenotypes, allowing rapid adaptation to different host environments [18]. This study shows that *S. pyogenes* also expressed PC, which was different for each strain, as assessed by the fluorescence intensity of flow cytometry. Furthermore, even with the same *emm* genotype, PC expression differed.

The M protein, a cell surface protein that is the major virulence of *S. pyogenes*, is encoded by the *emm* gene [19]. Classic M protein serological typing was largely replaced by sequence typing of the 5′ end of the *emm* gene in the late 1990s [20]. Kuhn et al. reported in a cohort study of 248 children in Canada that the most common *emm* genotype in patients with recurrent pharyngitis was *emm*12 (24.2%), followed by *emm*3 (18.2%), *emm1* (15.2%), and *emm*4 (12.1%) [21]. In addition, *emm*4, *emm*6, and *emm*75 strains invaded Detroit 562 cells significantly more than other genotypes [22]. Because of the small number of samples, it is impossible to compare. In this study, *emm*75 strains were 50% (4/8), while *emm*89, *emm*28, *em*m12, and *emm*11 strains were 12.5% each (1/8), and *emm*75 had a higher adhesiveness than the other *emm* genotypes.

High PC expression is thought to be highly pathogenic of *Streptococcus pneumoniae* and *Haemophilus influenzae* [23]. For instance, *Streptococcus pneumoniae* has been reported to cause more invasive infections in bacteria with a higher PC expression [24]. Andersson et al. reported that *Streptococcus pneumoniae* isolated from patients with acute otitis media adhered easily to cells, suggesting that the adhesive force is the virulence factor of *Streptococcus pneumoniae* [25]. Furthermore, strains isolated from the blood of patients with severe *S. pyogenes* infection have been reported to adhere more easily to cells [26]. In this study, PC expression and cell adhesion showed a positive correlation. These findings suggest that in *S. pyogenes*, higher PC expression makes it easier to adhere to cells and is more virulent.

This study showed that there was a significant negative correlation between TEPC-15 inhibitory effects and PC expression in *S. pyogenes*. Antibody responses to PC have been demonstrated in mouse experiments, and cells involved in the regulation of anti-PC antibody production have also been identified [27,28]. Kurono et al. reported that cell adhesion of *Streptococcus pneumoniae* and non-typeable *Haemophilus influenzae* was significantly reduced by treatment with a secretory IgA antibody [29]. In addition, the administration of PC to the nasal cavity of mice induced PC-specific IgA in mucosal secretions reacted with *Streptococcus pneumoniae* and non-typeable *Haemophilus influenzae* and increased clearance from the nasal cavity [30]. These findings supported the inhibitory effect of TEPC-15 on the adherence of these bacteria.

Furthermore, intracellular invasion assay experiments showed a significant negative correlation between TEPC-15 inhibitory effects and PC expression. In addition, PC expression and cell invasion showed a positive correlation. *S. pyogenes* invade host cells by internalization via phagosomes or endosomes [31,32]. Additionally, *S. pyogenes* form mature and antibiotic-resistant biofilms on physiologically relevant epithelial cells [33]. Ogawa et al. reported that more than half of the *emm*75 strains invaded cells and escaped the action of antibacterial agents even when penicillin was administered at 10 times the minimum inhibitory concentration [22]. In this study, *emm*75 strains had high PC expression and a large number of intracellular invasions. These findings suggested that PC expression is an important factor for bacteria that invades the cells.

PAF-R was inhibited on the cell surface to confirm the relationship between PC and PAF-R in adhesion to cells in *S. pyogenes*. Iuchi et al. reported that PAF-R expression in Detroit 562 cells was confirmed by FACS [23]. Cundell et al. reported that in *Streptococcus pneumoniae*, only highly pathogenic bacteria bound to PAF-R when adhering to cells [13]. Moreover, the expression of PAF-R is enhanced by viral antigens and contributes to the development of recurrent and persistent upper respiratory tract infections [18]. In this study, the inhibition of PAF-R with ABT-491 significantly inhibited cell adhesion and infiltration of bacteria with high PC expression rather than those with low PC expression. These results indicate that cellular PAF-R and bacterial PC are significantly involved in adhesion in *S. pyogenes* with high PC expression.

Even in bacteria with high PC expression, inhibition with TEPC-15 or ABT-491 did not suppress all adhesion to cells. This observation suggests that *S. pyogenes* involves PC and other adhesion factors. In fact, on the surface of *S. pyogenes*, there are various proteins, such as pili, SfbX and Lsp, which promote cell adhesion and colonization [10]. Furthermore, the administration of 100 μg/mL of TEPC-15 did not suppress cell adhesion. Weiser et al. reported three different levels of reactivity differences with TEPC-15 because of phase variation in the PC structure [34]. In addition, coating the PC epitope with TEPC-15 may lead to persistent infection [35]. Administering 100 μg/mL of TEPC-15 may influence avoid suppression of the adhesion by these factors.

Our study had some limitations. First, the number of bacteria used was small. However, by investigating the PC and *emm* genotypes together, we could examine from two aspects. Second, this study used cell lines derived from pharyngeal cancer. As these cells are of human origin, the results of this study were likely to be similar to those obtained using pharyngeal cells.

## 5. Conclusions

This study demonstrates that PC was expressed in *S. pyogenes*, and even if they had the same *emm* genotype, their remarks were different. It was found that PC and PAF-R are involved in cell adhesion and intracellular invasion of *S. pyogenes*.

## Figures and Tables

**Table 1:** PC expression and *emm* genotype on *Streptococcus pyogenes*.

Strain No.	PC Expression (MFI ± SD)	*emm* Genotype
1	64039.8 ± 25015.3	11
2	85783.1 ± 18703.1	12
3	99383.8 ± 13029.4	75
4	129073.1 ± 20423.0	75
5	165935.5 ± 28172.2	28
6	183695.3 ± 39675.8	89
7	247045.8 ± 33048.4	75
8	307203.2 ± 65813.9	75

MFI, mean fluorescence intensity (*n* = 5); SD, standard deviation; PC, phosphorylcholine.

## Data Availability

Not applicable.

## References

[1] Sakata H., Sato Y., Toyonaga Y., Hanaki H. (2017). Drug-Resistant Pathogen Surveillance Group in Pediatric Infectious Disease Serotype replacement of Streptococcus pneumoniae due to seven-valent pneumococcal conjugate vaccine in Japan. Pediatr. Int..

[2] Turk D.C. (1984). The pathogenicity of Haemophilus influenzae. J. Med. Microbiol..

[3] Luca-Harari B., Darenberg J., Neal S., Siljander T., Strakova L., Tanna A., Creti R., Ekelund K., Koliou M., Tassios P.T. (2009). Clinical and Microbiological Characteristics of Severe *Streptococcus pyogenes* Disease in Europe. J. Clin. Microbiol..

[4] Elodie R., Philippe A.G., Guillaume O., Etienne C., Laetitia E., Chouzenoux S., Weill B., Plainvert C., Poyart C., Batteux F. (2016). Superoxide anions produced by *Streptococcus pyogenes* group A-stimulated keratinocytes are responsible for cellular necrosis and bacterial growth inhibition. Innate Immun..

[5] Lepoutre A., Doloy A., Bidet P., Leblond A., Perrocheau A., Bingen E., Trieu-Cuot P., Bouvet A., Poyart C., Levy-Bruhl D. (2011). Epidemiology of Invasive *Streptococcus pyogenes* Infections in France in 2007. J. Clin. Microbiol..

[6] Lamagni T., Neal S., Keshishian C., Hope V., George R., Duckworth G., Vuopio-Varkila J., Efstratiou A. (2008). Epidemic of severe *Streptococcus pyogenes* infections in injecting drug users in the UK, 2003–2004. Clin. Microbiol. Infect..

[7] Seham A.S., Abdul-Raouf A.M., Sara H., Ahmed H.M., Gamal E. (2019). biological characterization and inhibition of *Streptococcus pyogenes* ZUH1 causing chronic cystitis by crocus sativus methanol extract, bee honey alone or in combination with antibiotics: An in vitro study. Molecules.

[8] Iuchi H., Ohori J., Kyutoku T., Ito K., Kawabata M. (2020). Inhibitory effects of 2-methacryloyloxyethyl phosphorylcholine polymer on the adherence of bacteria causing upper respiratory tract infection. J. Oral Microbiol..

[9] Wang B., Ruiz N., Pentland A., Caparon M. (1997). Keratinocyte pro-inflammatory responses to adherent and nonadherent group A strepto-cocci. Infect. Immun..

[10] Stephan B., Timothy C.B., Tania R.H., Manfred R., Mark J.W. (2016). *Streptococcus pyogenes* adhesion and colonization. FEBS Lett..

[11] Chang H., Shen X., Huang G., Fu Z., Zheng Y., Wang L., Li C., Liu L., Shen Y., Liu X. (2011). Molecular analysis of *Streptococcus pyogenes* strains isolated from Chinese children with pharyngitis. Diagn. Microbiol. Infect. Dis..

[12] Chuang I., Van Beneden C., Beall B., Schuchat A. (2002). Population-based surveillance for postpartum invasive group a strepto-coccus infections, 1995–2000. Clin. Infect. Dis..

[13] Cundell D.R., Gerard N.P., Gerard C., Idanpaan-Heikkila I., Tuomanen E. (1995). *Streptococcus pneumoniae* anchor to activated human cells by the receptor for platelet-activating factor. Nature.

[14] Swords W.E., Buscher B.A., Ver Steeg Ii K., Preston A., Nichols W.A., Weiser J.N., Gibson B.W., Apicella M.A. (2000). Non-typeable *Haemophilus influenzae* adhere to and invade human bronchial epithelial cells via an interaction of lipooligo-saccharide with the PAF receptor. Mol. Microbiol..

[15] Courtney H.S., Ofek I., Penfound T., Nizet V., Pence M.A., Kreikemeyer B., Podbielski A., Hasty D.L., Dale J.B. (2009). Relationship between expression of the family of M proteins and lipoteichoic acid to hydrophobicity and biofilm formation in *Streptcoccus pyogenes*. PLoS ONE.

[16] Tomasz A. (1967). Choline in the Cell Wall of a Bacterium: Novel Type of Polymer-Linked Choline in Pneumococcus. Science.

[17] Poxton I.R., Tarelli E., Baddiley J., Watson M.J., Brundish D.E., Heckels J.E., Lambert P.A., Wicken A. (1978). The structure of C-polysaccharide from the walls of *Streptococcus pneumoniae*. Biochem. J..

[18] Sarah E.C., Julian S., Jianjun L., Tracey A.Z., Jeffrey N.W. (2012). Phosphorylcholine allows for evasion of bactericidal antibody by Haemophilus influenzae. PLoS Pathog..

[19] Syrogiannopoulos G.A., Grivea I.N., Al-Lahham A., Panagiotou M., Tsantouli A.G. (2013). Michoula Ralf René Reinert AN, van der Linden, M. Seven-year surveillance of *emm* types of pediatric Group A streptococcal pharyngitis isolates in Western Greece. PLoS ONE.

[20] Steer A.C., Law I., Matatolu L., Beall B.W., Carapetis J. (2009). Global *emm* type distribution of group A streptococci: Systematic review and implications for vaccine development. Lancet Infect. Dis..

[21] Kuhn S.M., Preiksaitis J., Tyrrell G.J., Jadavji T., Church D., Dele Davies H. (2001). Evaluation of Potential Factors Contributing to Microbiological Treatment Failure in *Streptococcus Pyogenes* Pharyngitis. Can. J. Infect. Dis..

[22] Ogawa T., Terao Y., Okuni H., Ninomiya K., Sakata H., Ikebe K., Maeda Y., Kawabata S. (2011). Biofilm formation or internal-ization into epithelial cells enable *Streptococcus pyogenes* to evade antibiotic eradication in patients with pharyngitis. Microb. Pathog..

[23] Iuchi H., Ohori J., Kyutoku T., Ito K., Kurono Y. (2019). Role of phosphorylcholine in Streptococcus pneumoniae and nontypeable Haemophilus influenzae adherence to epithelial cells. Auris Nasus Larynx.

[24] Briles D.E., Forman C., Crain M. (1992). Mouse antibody to phosphocholine can protect mice from infection with mouse-virulent human isolates of *Streptococcus pneumoniae*. Infect. Immun..

[25] Andersson B., Nylén O., Peterson C.M., Svanborg-Edén C. (1980). Attachment of *Streptococcus pneumoniae* to human pharyngeal epithelial cells in vitro. Ann. Otol. Rhinol. Laryngol. Suppl..

[26] Edwards M.L., Fagan P.K., Smith-Vaughan H., Currie B.J., Sriprakash K.S. (2003). Strains of *Streptococcus pyogenes* from Severe Invasive Infections Bind HEp2 and HaCaT Cells More Avidly than Strains from Uncomplicated Infections. J. Clin. Microbiol..

[27] Kohker H. (1975). The response to phosphorylcholine: Dissecting and immune response. Transplant. Rev..

[28] Mond J.J., Lieberman R., Inman J.K., Mosier D.E., Paul W.E. (1977). Inability of mice with a defect in B lymphocyte maturation to respond to phosphorylcholine on immunogenic carriers. J. Exp. Med..

[29] Kurono Y., Shigemi H., Shimamura K., Mogi G. (1991). Inhibition of Bacterial Adherence by Nasopharyngeal Secretions. Ann. Otol. Rhinol. Laryngol..

[30] Tanaka N., Fukuyama S., Fukuiwa T., Kawabata M., Sagara Y., Ito H.-O., Miwa Y., Nagatake T., Kiyono H., Kurono Y. (2007). Intranasal immunization with phosphorylcholine induces antigen specific mucosal and systemic immune responses in mice. Vaccine.

[31] Molinari G., Rohde M., Guzman C.A., Chhatwal G.S. (2000). Two distinct pathways for the invasion of Streptococcus pyogenes in non-phagocytic cells. Cell. Microbiol..

[32] Tjelle T.E., Løvdal T., Berg T. (2000). Phagosome dynamics and function. BioEssays.

[33] Feiruz A., Yashuan C., Maria B., Kristian R., Anders P.H. (2020). A role of epithelial cells and virulence factors in biofilm formation by Streptococcus pyogenes in vitro. Infect. Immun..

[34] Weiser J.N., Shchepetov M., Chong S.T. (1997). Decoration of lipopolysaccharide with phosphorylcholine: A phase-variable charac-teristic of Haemophilus influenzae. Infect. Immun..

[35] Sarah E.C., Jeffrey N.W. (2013). Microbial modulation of host immunity with the small molecule phosphorylcholine. Infect. Immun..

